# Blood transfusions increase the risk for venous thromboembolism events following total joint arthroplasty

**DOI:** 10.1038/s41598-021-00263-0

**Published:** 2021-10-28

**Authors:** Noam Shohat, Leanne Ludwick, Graham S. Goh, Matthew Sherman, Joseph Paladino, Javad Parvizi

**Affiliations:** 1 Rothman Orthopaedic Institute at Thomas Jefferson University, Philadelphia, PA USA; 2 Sackler Faculty of Medicine, Tel Aviv University, Ramat Aviv, Israel; 3 Rothman Orthopaedic Institute, 125 S 9th St. Ste 1000, Philadelphia, PA 19107 USA

**Keywords:** Orthopaedics, Outcomes research

## Abstract

The association between blood transfusions and thromboembolic events (VTE) following total joint arthroplasty (TJA) remains debatable. Using contemporary institutional data, this study aimed to determine whether blood transfusions increase the risk of VTE following primary and revision TJA. This was a single institution, retrospective cohort study. The clinical records of all patients (n = 34,824) undergoing primary and revision TJA between 2009 and 2020 were reviewed. Demographic variables, co-morbidities, type of chemoprophylaxis and intraoperative factors such as use of tranexamic acid were collected. Clinical notes, hospital orders, and discharge summaries were reviewed to determine if a patient received a blood transfusion. Comprehensive queries utilizing keywords for VTE were conducted in clinical notes, physician dictations, and patient-provider phone-call logs. Propensity score matching as well as adjusted mixed models were performed. After adjusting for various confounders, results from regression analysis showed a significant association between allogenic blood transfusions and risk for developing VTE following primary and revision TJA (OR 4.11, 95% CI 2.53–6.69 and OR 2.15, 95% CI 1.12–4.13, respectively). While this strong association remained significant for PE in both primary (p < 0.001) and revision (p < 0.001) matched cohorts, it was no longer statistically significant for DVT (p = 0.802 and p = 0.65, respectively). These findings suggest that the risk of VTE is increased by approximately three-folds when blood transfusions are prescribed. This association was mainly due to higher symptomatic PE events which makes it even more worrisome. Surgeons should be aware of this association, revisit criteria for blood transfusions and use all means available in the perioperative period to optimize the patients and avoid transfusion.

## Introduction

Venous thromboembolism (VTE), including deep venous thrombosis (DVT) and pulmonary embolism (PE), is a dreadful complication associated with total joint arthroplasty (TJA). While several risk factors for the development of VTE have been established^1,2^, others are still widely debated^2,3^. One potentially modifiable risk factor that has gained attention due to its common and unrestricted use is allogeneic blood transfusion^4–6^.

The demand for blood products is on the rise worldwide resulting in significant financial and health related costs^7–9^. It is estimated that more than 400 million whole blood and red blood cell (RBC) units are transfused annually in the United States^10^. Physicians in general, and orthopedic surgeons in particular, tend to overprescribe blood transfusions^11–14^. In fact, Total hip (THA) and knee (TKA) arthroplasties represent the number one reason for allogeneic blood transfusion in patients undergoing elective surgery, accounting for nearly 10% of all hospital transfused red blood cell units^12,13^. Often times the amount of blood transfused, does not clearly correlate with the blood volume that was lost during surgery, and is often times to the detriment of patient health^15,16^.

Several attempts were made to try and shed light on whether blood transfusions lead to increased risk for VTE. A review of the current literature shows that registry data has consistently demonstrated an association between blood transfusions and VTE. However, all published studies to date have utilized the same National Surgical Quality Improvement Program (NSQIP) database^4,5^, rendering them susceptible to inherent limitations associated with registry data^17,18^. By contrast, several institutional studies, including one published from our institution, have failed to demonstrate a clear independent association^6,19^. However, these studies included only primary TJA, they suffered from sample size limitations and failed to account for the change in practice patterns that have been introduced over the past decade, such as the use of tranexamic acid (TXA) and transition to aspirin as the main chemoprophylaxis agent in TJA. Given the recent advances in perioperative care as well as the lack of consensus in the literature, an updated investigation of the impact of blood transfusions on the risk of VTE following primary and revision TJA is therefore warranted.

Using contemporary institutional data, the aim of this study was to assess whether blood transfusion increases the risk of VTE following primary and revision TJA.

## Methods

A single institution, retrospective cohort study was conducted following approval by the institutional review board of Thomas Jefferson University with a waiver of informed consent. This study was performed in accordance with relevant guidelines and regulations. The clinical records of 39,948 consecutive patients who underwent a primary or revision THA or TKA from January 2009 to October 2020 were reviewed. Strengthening the reporting of observational studies in epidemiology (STROBE) reporting guidelines were followed throughout the data collection process^20^. Patients for whom transfusion data was not available were excluded. The final cohort consisted of 34,824 patients with a minimum follow-up of 90 days.

Age, sex, race, body mass index (BMI), Charlson Comorbidity Index (CCI), Elixhauser Comorbidity Index (ECI), and American Society of Anesthesiologists (ASA) classification were collected through hospital medical records and International Classification of Diseases (ICD) codes. Clinical notes, hospital orders, and discharge summaries were reviewed to record the type of VTE prophylaxis prescribed to each patient postoperatively. Operative details including the specific joint operated on, laterality, operative time, use of cement, TXA administration, and intraoperative blood transfusions were also collected from operative reports and anesthesia case logs.

**Figure 1:**
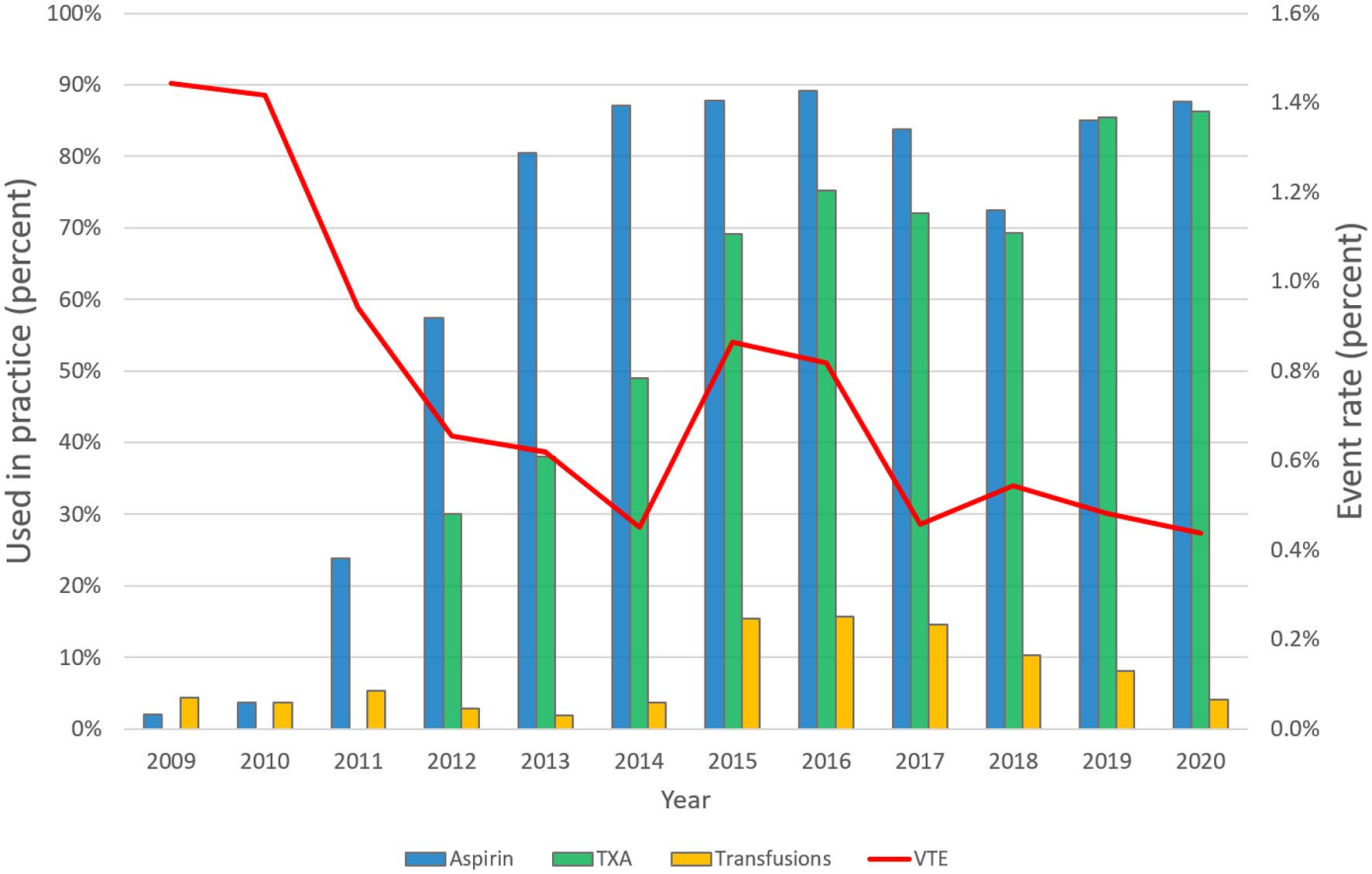
Relevant changes in practice over the course of the study. Bars reflect changes in aspirin and tranexamic usage as well as transfusion rates. The line represents symptomatic venous thromboembolism rates.

Over the course of this study, our institution underwent a transition from the use of warfarin to aspirin as the main method for chemoprophylaxis of VTE. Warfarin remained the treatment of choice for high risk patients^2^. Patients receiving warfarin had their INR monitored by either their personal physician or a warfarin clinic. Patients receiving aspirin were either administered regular dose (325 mg) or low dose (81 mg) twice daily. VTE prophylaxis with either agent was continued for 4 to 6 weeks postoperatively. Additional relevant change in practice during the course of the study included transition to routine use of tranexamic acid infusion during surgery (Fig. 1).

The main exposure variable was the use of allogeneic blood transfusion (packed red blood cells). This was divided into 3 distinct categories: no transfusion, 1 transfusion unit and 2 or more transfusion units. The latter two categories were grouped together for the main analysis, but analyzed individually as a subgroup in adjusted models. The primary outcome was the first occurrence of a symptomatic DVT or PE within 90 days of surgery. Symptomatic VTE occurring within 90 days of the operation were identified from medical records through ICD 9 and 10 codes (Supplementary Table S1). To enhance the capture rate, comprehensive queries utilizing keywords for DVT and PE were conducted in clinical notes, physician dictations, and patient-provider phone-call logs (Supplementary Table S2). Notes containing a keyword for DVT (n = 44,752) and PE (n = 14,878) were isolated and manually reviewed. All readmissions within 90 days were also reviewed to detect any uncaptured VTE event. Patients that had a documented diagnosis, confirmatory study (ultrasound for DVT or CT scan for PE), or received treatment for VTE were considered to have met the primary endpoint. For events that were diagnosed in an outside facility for which an objectively confirmed study was not readily available to review, diagnosis relied on a documented note reporting a confirmatory study as well as a symptomatic event that received treatment. To avoid inclusion of clinically insignificant clots (unimportant or small, possibly muscle vein clots), cases were excluded if they were specifically documented as asymptomatic or not mandating anticoagulation treatment.

R Studio (version 3.6.3, Vienna, Austria) was used for the statistical analyses^21^. The level of statistical significance was defined as a p-value of less than under 0.05. To minimize the loss of power and decrease bias owing to exclusion of cases that were missing 1 or more covariates, missing covariates were imputed 10 times using the multivariate sequential regression approach. First, univariate comparisons between patients who did not receive blood transfusion compared to those who received one or more transfusions were performed, using chi-square for dichotomous variables, and T-test for continuous variables. Second, an unadjusted followed by adjusted logistic regression analysis looking at mixed models were performed with VTE as the dependent outcome. For these analyses, blood transfusion was broken down into three distinct categories (no transfusion, 1 transfusion, and 2 or more transfusions). Significant outcomes in the univariate that were included in the regression analysis included blood transfusion, age, BMI, sex, ASA score, CCI score, joint, unilateral vs. bilateral, and use of TXA. The patient identification number was used as the random term to account for patients who underwent multiple procedures to avoid bias from unmeasurable risk factors. Third, to account for significant differences between the non-transfusion and transfusion groups, the groups were matched for age, BMI, sex, all comorbidities in the cohort (see Table 1), joint, unilateral vs. bilateral, primary vs. revision, as well as the use of cement and TXA using propensity score matching.

**Table 1:** Patient characteristics, comorbidities, operative factors and type of VTE prophylaxis for the primary TJA cohort and their VTE rates.

	No transfusion (n = 28,529)	Any transfusion (n = 956)	P-value
**Demographics**			
Age, year (mean,SD)	63.7 (10.5)	67.9 (12.4)	< 0.001
Sex, female	15,491 (54.3%)	559 (58.5%)	0.012
BMI, Kg/M^2^ (mean,SD)	29.9 (5.36)	28.9 (5.74)	< 0.001
**Co-morbidities**			
CCI (mean,SD)	0.39 (0.84)	1.16 (1.67)	< 0.001
History of VTE	926 (3.25%)	77 (8.05%)	< 0.001
CHF	455 (1.59%)	95 (9.94%)	< 0.001
CPD	3157 (11.1%)	171 (17.9%)	< 0.001
CVD	318 (1.11%)	44 (4.60%)	< 0.001
Dementia	42 (0.15%)	6 (0.63%)	0.004
DM	1390 (4.87%)	93 (9.73%)	< 0.001
Hemiparesis	15 (0.05%)	2 (0.21%)	0.104
Malignancy			
Non-metastatic	243 (0.85%)	26 (2.72%)	< 0.001
Metastatic	41 (0.14%)	20 (2.09%)	
MI	965 (3.38%)	65 (6.80%)	< 0.001
Liver disease			
Mild	265 (0.93%)	25 (2.62%)	< 0.001
Moderate/severe	11 (0.04%)	6 (0.63%)	< 0.001
PUD	82 (0.29%)	23 (2.41%)	< 0.001
PVD	388 (1.36%)	41 (4.29%)	< 0.001
Renal failure	571 (2.00%)	107 (11.2%)	< 0.001
Rheumatic disease	856 (3.00%)	55 (5.75%)	< 0.001
**Operative factors**			
Joint, knee	13,750 (48.2%)	439 (45.9%)	0.176
Bilateral surgery	1724 (6.04%)	95 (9.94%)	< 0.001
Tranexamic acid	13,380 (46.9%)	386 (40.4%)	< 0.001
Operative time (mean,SD)	72.2 (26.6)	98.5 (55.5)	< 0.001
Cement used	11,273 (39.5%)	403 (42.2%)	0.108
**Anticoagulation**			
Aspirin	17,653 (61.9%)	598 (62.6%)	0.697
Other	10,876 (38.1%)	358 (37.4%)	
**Symptomatic VTE**	198 (0.69%)	31 (3.24%)	< 0.001
Pulmonary Emboli	122 (0.43%)	25 (2.62%)	< 0.001
Deep Vein Thrombosis	96 (0.34%)	9 (0.94%)	0.007

*BMI* Body Mass Index, *Kg* kilogram, *M* meter, *CCI* Charlson Comorbidity Index, *CHF* congestive heart failure, *CPD* cardiopulmonary disease, *CVD* cardiovascular disease, *DM* diabetes mellitus, *MI* myocardial infraction, *PUD* peptic ulcer disease, *PVD* peripheral vascular disease, *VTE* venous thromboembolism event.

## Results

Of the 34,824 patients that were eligible for analysis, 2063 (5.9%) received one or more units blood transfusions. There were statistically significant differences between the non-transfusion and transfusion groups in both the primary and revision cohort. Patients who received a blood transfusion were older and had more comorbidities and being older. There were also marked differences in operative factors between the 2 groups (Tables 1 and 2).

**Table 2:** Patient characteristics, comorbidities, operative factors and type of VTE prophylaxis for the revision TJA cohort and their VTE rates.

	No transfusion (n = 4232)	Any transfusion (n = 1107)	P-value
**Demographics**			
Age, year (mean,SD)	63.0 (12.8)	66.5 (12.5)	< 0.001
Sex, female	2169 (51.3%)	595 (53.7%)	0.148
BMI, Kg/M^2^ (mean,SD)	30.1 (6.18)	30.4 (6.90)	0.422
**Co-morbidities**			
CCI (mean,SD)	0.55 (1.10)	1.16 (1.62)	< 0.001
History of VTE	284 (6.71%)	102 (9.21%)	0.005
CHF	125 (2.95%)	101 (9.12%)	< 0.001
CPD	524 (12.4%)	205 (18.5%)	< 0.001
CVD	66 (1.56%)	49 (4.43%)	< 0.001
Dementia	6 (0.14%)	26 (2.35%)	< 0.001
DM	169 (3.99%)	82 (7.41%)	< 0.001
Hemiparesis	8 (0.19%)	6 (0.54%)	0.051
Malignancy			
Non-metastatic	58 (1.37%)	22 (1.99%)	0.069
Metastatic	28 (0.66%)	13 (1.17%)	
MI	196 (4.63%)	89 (8.04%)	< 0.001
Liver disease			
Mild	32 (0.76%)	25 (2.26%)	< 0.001
Moderate/severe	1 (0.02%)	10 (0.90%)	< 0.001
PUD	17 (0.40%)	9 (0.81%)	0.132
PVD	69 (1.63%)	46 (4.16%)	< 0.001
Renal failure	158 (3.73%)	142 (12.8%)	< 0.001
Rheumatic disease	217 (5.13%)	86 (7.77%)	< 0.001
**Operative factors**			
Joint, knee	1757 (41.5%)	353 (31.9%)	< 0.001
Bilateral surgery	40 (0.95%)	12 (1.08%)	0.805
Tranexamic acid	1224 (28.9%)	360 (32.5%)	0.022
Operative time (mean,SD)	120 (50.0)	149 (75.6)	< 0.001
Cement used	1840 (43.5%)	478 (43.2%)	0.885
**Anticoagulation**			
Aspirin	1790 (42.3%)	553 (50.0%)	< 0.001
Other	2442 (57.7%)	554 (50.0%)	
**Symptomatic VTE**	49 (1.16%)	37 (3.34%)	< 0.001
Pulmonary emboli	8 (0.19%)	12 (1.08%)	< 0.001
Deep vein thrombosis	43 (1.02%)	28 (2.53%)	< 0.001

*BMI* Body Mass Index, *Kg* kilogram, *M* meter, *CCI* Charlson Comorbidity Index, *CHF* congestive heart failure, *CPD* cardiopulmonary disease, *CVD* cardiovascular disease, *DM* diabetes mellitus, *MI* myocardial infraction, *PUD* peptic ulcer disease, *PVD* peripheral vascular disease, *VTE* venous thromboembolism event.

### Primary TJA

**Figure 2:**
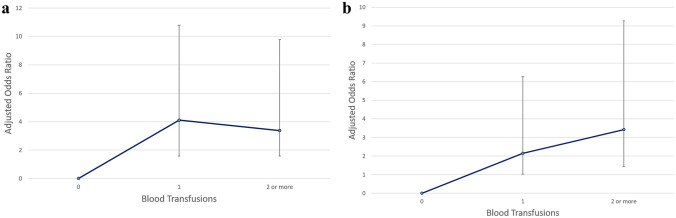
Adjusted odds ratios (ORs) of symptomatic venous thromboembolism with increased number of transfusions versus no transfusion in the primary cohort (**a**) and revision cohort (**b**). *Bars reflecting 95% confidence interval.

In the unmatched cohort, the rate of symptomatic VTE was 0.69% (198/28,529) in the non-transfusion group compared to 3.24% (31/956) in the transfusion group (p < 0.001). This association remained significant for both PE and DVT separately (Table 1). Regression analysis showed a significant association between 1 blood transfusion (OR 4.11, 95% CI 2.53–6.69, p < 0.001) and 2 or more blood transfusions (OR 3.38, 95% CI 1.79–6.39, p < 0.001) with risk for developing VTE (Fig. 2a). Others factors that were significantly associated with VTE in the regression analysis included older age, higher BMI, TKA (as opposed to THA), bilateral arthroplasties, and non-aspirin prophylaxis (Table 3).

**Table 3:** Logistic mixed model regression evaluating the association between 1 blood transfusion, or 2 or more (with zero transfusions as the reference) and risk for VTE in the primary TJA cohort.

Variable	Estimate	Odds ratio	Lower 95	Upper 95	P Value
**Blood transfusion**					
1	1.41	4.11	2.53	6.69	< 0.001
2 or more	1.22	3.38	1.79	6.39	< 0.001
Age	0.02	1.02	1.01	1.04	0.001
BMI, Kg/M^2^	0.05	1.05	1.03	1.07	< 0.001
Male	0.25	1.29	0.997	1.66	0.053
Aspirin	− 0.43	0.65	0.48	0.89	0.006
CCI	0.10	1.11	0.997	1.24	0.056
Hip	− 0.74	0.48	0.36	0.64	< 0.001
Bilateral	0.75	2.11	1.44	3.11	< 0.001
TXA	− 0.33	0.72	0.51	1.01	0.056
Op time	0.002	1.002	0.998	1.01	0.307

*BMI* Body Mass Index, *Kg* kilogram, *M* meter, *CCI* Charlson Comorbidity Index, *TXA* tranexamic acid.

The propensity score matched cohort comprised 956 patients per group. Both groups showed no significant difference in demographic variables, comorbidities, operative factors or chemoprophylaxis medications (Table 4). In this matched cohort, the association between blood transfusions with the risk for developing VTE remained significant (p = 0.002) and was 3 times higher in patients who received blood transfusion (3.24%) compared to those who did not (1.05%). While this association remained significant for PE (2.62% vs. 0.31%, p < 0.001) it was no longer statistically significant for DVT (0.94% vs. 0.73%, p = 0.802).

**Table 4:** Patient characteristics, comorbidities, operative factors and type of VTE prophylaxis for the matched primary TJA cohort and VTE rates.

	No transfusion (n = 956)	Any transfusion (n = 956)	P-value
**Demographics and habits**			
Age, year (mean, SD)	68.4 (10.3)	67.9 (12.4)	0.980
Sex, female	539 (56.4%)	559 (58.5%)	0.380
BMI, Kg/M^2^ (mean, SD)	28.8 (5.19)	28.9 (5.74)	0.955
**Co-morbidities**			
CCI (mean,SD)	1.25 (1.81)	1.16 (1.67)	0.533
History of VTE	79 (8.26%)	77 (8.05%)	0.933
CHF	108 (11.3%)	95 (9.94%)	0.373
CPD	184 (19.2%)	171 (17.9%)	0.480
CVD	54 (5.65%)	44 (4.60%)	0.351
Dementia	5 (0.52%)	6 (0.63%)	1.000
DM	112 (11.7%)	93 (9.73%)	0.183
Hemiparesis	2 (0.21%)	2 (0.21%)	1.000
Malignancy			
Non-metastatic	30 (3.14%)	26 (2.72%)	0.863
Metastatic	20 (2.09%)	20 (2.09%)	
MI	76 (7.95%)	65 (6.80%)	0.382
Liver disease			
Mild	22 (2.30%)	25 (2.62%)	0.768
Moderate/severe	6 (0.63%)	6 (0.63%)	1.000
PUD	17 (1.78%)	23 (2.41%)	0.424
PVD	47 (4.92%)	41 (4.29%)	0.585
Renal failure	104 (10.9%)	107 (11.2%)	0.884
Rheumatic disease	50 (5.23%)	55 (5.75%)	0.688
**Operative factors**			
Joint, knee	443 (46.3%)	439 (45.9%)	0.891
Bilateral surgery	109 (11.4%)	95 (9.94%)	0.336
Tranexamic acid	380 (39.7%)	386 (40.4%)	0.815
Operative time (mean, SD)	93.9 (55.0)	98.5 (55.5)	0.074
Cement used	397 (41.5%)	403 (42.2%)	0.815
**Anticoagulation**			
Aspirin	611 (63.9%)	598 (62.6%)	0.569
Other	345 (36.1%)	358 (37.4%)	
**Symptomatic VTE**	10 (1.05%)	31 (3.24%)	0.002
Pulmonary emboli	3 (0.31%)	25 (2.62%)	< 0.001
Deep vein thrombosis	7 (0.73%)	9 (0.94%)	0.802

*BMI* Body Mass Index, *Kg* kilogram, *M* meter, *CCI* Charlson Comorbidity Index, *CHF* congestive heart failure, *CPD* cardiopulmonary disease, *CVD* cardiovascular disease, *DM* diabetes mellitus, *MI* myocardial infraction, *PUD* peptic ulcer disease, *PVD* peripheral vascular disease, *VTE* venous thromboembolism event.

**The groups were matched for age, BMI, sex, all comorbidities in the cohort (see Table 1), joint, unilateral vs. bilateral, as well as the use of cement and TXA.

### Revision TJA

In the unmatched cohort, the rate of symptomatic VTE was 1.16% (49/4232) in the non-transfusion group compared to 3.34% (37/1107) in the transfusion group (p < 0.001). This association remained significant for both PE and DVT separately (Table 2). Regression analysis showed a significant association between 1 blood transfusion (OR 2.15, 95% CI 1.12–4.13, p = 0.021) and 2 or more blood transfusions (OR 3.42, 95% CI 1.99–5.85, p < 0.001) with risk for developing VTE (Fig. 2b). The only others factor that were significantly associated with VTE in the regression analysis was non-aspirin prophylaxis (Table 5).

**Table 5:** Logistic mixed model regression evaluating the association between 1 blood transfusion, or 2 or more (with zero transfusions as the reference) and risk for VTE in the revision TJA cohort.

Variable	Estimate	Odds ratio	Lower 95	Upper 95	P value
**Blood transfusion**					
1	0.77	2.15	1.12	4.13	0.021
2 or more	1.23	3.42	1.99	5.85	< 0.001
Age	0.02	1.02	0.998	1.04	0.070
BMI, Kg/M^2^	0.01	1.01	0.98	1.04	0.578
Male	0.27	1.31	0.85	2.02	0.221
Aspirin	− 1.40	0.25	0.13	0.48	< 0.001
CCI	0.08	1.08	0.95	1.23	0.216
Hip	− 0.37	0.69	0.44	1.08	0.105
TXA	− 0.43	0.65	0.32	1.34	0.244
Op time	0.001	1.00	0.997	1.004	0.617

*BMI* Body Mass Index, *Kg* kilogram, *M* meter, CCI Charlson Comorbidity Index, *TXA* tranexamic acid.

The propensity score matched cohort comprised 1107 patients per group. The only factors that remained significantly different between the 2 groups after matching were dementia and liver disease (Table 6). In this matched cohort, the association between blood transfusions with the risk for developing VTE remained significant (p = 0.003) and was 2.5 times higher in patients who received blood transfusion (3.34%) compared to those who did not (1.36%). While this association remained significant for PE (1.08% vs. 0.09%, p = 0.005) it was no longer statistically significant for DVT (1.36% vs. 2.53%, p = 0.065).

**Table 6:** Patient characteristics, comorbidities, operative factors and type of VTE prophylaxis for the matched revision TJA cohort and VTE rates.

	No transfusion (n = 1107)	Any transfusion (n = 1107)	P-value
**Demographics and habits**			
Age, year (mean, SD)	66.7 (11.4)	66.5 (12.5)	0.782
Sex, female	593 (53.6%)	595 (53.7%)	0.966
BMI, Kg/M^2^ (mean, SD)	30.7 (6.65)	30.4 (6.90)	0.217
**Co-morbidities**			
CCI (mean, SD)	1.10 (1.53)	1.16 (1.62)	0.499
History of VTE	100 (9.03%)	102 (9.21%)	0.941
CHF	91 (8.22%)	101 (9.12%)	0.497
CPD	214 (19.3%)	205 (18.5%)	0.664
CVD	39 (3.52%)	49 (4.43%)	0.328
Dementia	6 (0.54%)	26 (2.35%)	0.001
DM	80 (7.23%)	82 (7.41%)	0.935
Hemiparesis	80 (7.23%)	82 (7.41%)	0.751
Malignancy			
Non-metastatic	24 (2.17%)	22 (1.99%)	0.727
Metastatic	17 (1.54%)	13 (1.17%)	
MI	81 (7.32%)	89 (8.04%)	0.576
Liver disease			
Mild	24 (2.17%)	25 (2.26%)	1.000
Moderate/severe	1 (0.09%)	10 (0.90%)	0.016
PUD	7 (0.63%)	9 (0.81%)	0.802
PVD	35 (3.16%)	46 (4.16%)	0.258
Renal failure	127 (11.5%)	142 (12.8%)	0.362
Rheumatic disease	100 (9.03%)	86 (7.77%)	0.319
**Operative factors**			
Joint, knee	392 (35.4%)	353 (31.9%)	0.087
Bilateral surgery	14 (1.26%)	12 (1.08%)	0.844
Tranexamic acid	392 (35.4%)	360 (32.5%)	0.164
Operative time (mean,SD)	144 (64.6)	149 (75.6)	0.381
Cement used	513 (46.3%)	478 (43.2%)	0.146
**Anticoagulation**			
Aspirin	572 (51.7%)	553 (50.0%)	0.444
Other	535 (48.3%)	554 (50.0%)	
**Symptomatic VTE**	15 (1.36%)	37 (3.34%)	0.003
Pulmonary emboli	1 (0.09%)	12 (1.08%)	0.005
Deep vein thrombosis	15 (1.36%)	28 (2.53%)	0.065

*BMI* Body Mass Index, *Kg* kilogram, *M* meter, *CCI* Charlson Comorbidity Index, *CHF* congestive heart failure, *CPD* cardiopulmonary disease, *CVD* cardiovascular disease, *DM* diabetes mellitus, *MI* myocardial infraction, *PUD* peptic ulcer disease, *PVD* peripheral vascular disease, *VTE* venous thromboembolism event.

**The groups were matched for age, BMI, sex, all comorbidities in the cohort (see Table 1), joint, unilateral vs. bilateral, as well as the use of cement and TXA.

## Discussion

This is the largest single institution study to date to evaluate the association between blood transfusions and the risk for subsequent symptomatic VTE following primary and revision TJA. Using a contemporary cohort to account for major advances in perioperative care over the past decade, our findings suggest that an independent, dose-dependent association between allogeneic blood transfusions and the risk for subsequent postoperative VTE exists. Given the three-fold increase in VTE risk following a blood transfusion, the present findings help to raise awareness about this important association and underscore the need for stringent perioperative protocols to ration the use of blood transfusions following TJA.

The biological mechanisms substantiating the increased thrombotic risk following blood transfusion have been well described^22,23^. It is important to acknowledge that increased blood viscosity is an established risk factor for thrombosis and the shape, deformability, and viscoelastic properties of red blood cells make them the primary determinants of viscosity^24–26^. Red blood cells also bind to fibrinogen, and this interaction is a key mediator of blood viscosity^26^. In vitro and mouse models imply direct and indirect complex biochemical mechanisms by which red blood cells contribute to thrombus initiation and propagation^27,28^. One suggested mechanism is through modulation of the inflammatory cascade^29^. This could result from a direct immune response to the blood transfusion or due to oxidative stress related to the storage of red blood cells and consequently surface damage resulting in a pro-coagulative state^27,30^. Other studies suggest platelet over activity as a possible causative factor^23^. Elevated hematocrit increases the interaction and adhesion of platelets to thrombi and platelets isolated from RBC-transfused patients show significantly enhanced aggregation^31^. This growing body of molecular evidence corresponds to the clinical findings of our study.

Recently published registry data, all from the NSQIP database, have suggested an association between blood transfusions and VTE following orthopedic procedures^4,5^. Goel et al. analyzed 750,937 patients from this database, of which 153,320 underwent orthopedic surgery. The results of their subgroup analysis showed an adjusted OR of 1.7 (95% CI 1.5–2.0) for developing VTE following blood transfusion. Consistent with these findings, Acuña et al. utilized the same database and timeframe to examine the association in 333,463 patients undergoing TKA^5^. While they found an initial association between perioperative blood transfusions and DVT (adjusted OR 1.32, 95% CI 1.14–1.53), their propensity score model failed to confirm this association. Notwithstanding, major limitations of the abovementioned studies that are common to registry-based data are the inaccuracies in data input as well as inability to control for inherent biases arising from wide practice variations and unmeasurable confounders^17,18^. While they attempted to adjust for demographic and co-morbidities, other confounders that have been suggested to play major role in the development of VTE, such as a history of previous VTE, operative factors, type of chemoprophylaxis and other contemporary changes in practice including the use of TXA could not be accounted for as these variables are not well documented in national registries. Using contemporary data derived from a single institution to circumvent the inherent biases from unmeasurable confounders, the present findings echo that of previous studies. All efforts were made to isolate and determine the independent association between blood transfusion and VTE using adjusted models and propensity score matching, and our results reinforce the notion that blood transfusions increase the risk for VTE following both primary and revision TJA. Propensity score analysis results suggested that this association is mainly true for PE events as the association with DVT lost statistical significance in the matched cohort.

The current findings were contradictory to those published from our own institution previously^6^, which is a testament to the immense difficulty in isolating a single variable association when the event rate is low and many confounding variables exist. While the study by Jackson et al. evaluated a large number of 29,000 TJA, the event rate was only 1.04% (the number of VTEs in the transfused group), possibly rendering the study underpowered to detect an effect size that may be still clinically meaningful. This limitation was further highlighted when an admirable attempt was made to adjust for a large number of variables based on a validated VTE risk score, which may in turn have resulted in overadjustment^32^. Another plausible reason for the discrepancy between our two studies could be the difference in time period between the study cohorts. Jackson et al. included all primary TJA patients operated since 2000 and did not include the last 6 years of practice in their analysis. Consequently, only 20% of our cohort had overlapped with that of the previous study. Changes in the perioperative care including early mobility, the use of TXA, more aggressive rehabilitation, same-day discharge, and transition to aspirin as the chemoprophylactic agent of choice could not be accounted for in the previous publication and may have confounded the results as well.

The annual total units of blood transfused in U.S. hospitals has increased from 15 to 21 million in a short span of 5 years^33^. Frank et al. examined the medical records of more than 48,000 surgical patients over an 18-month period and found that 2,981 patients (6.2 percent) were given blood transfusions during surgery^13^. Interestingly, the authors noted wide variations in blood transfusion prescribing patterns among surgeons and anesthesiologists. For instance, patients undergoing cardiac surgeries received blood at much lower trigger points compared to patients having other surgeries, whereas patients undergoing orthopedic surgery received blood at higher trigger points (often at or above 10 g per deciliter). Moreover, the amount of blood transfused did not correlate the clinical status of the patient or the degree of blood loss typically expected during each type of surgery^15,16^. In addition, orthopedic surgeons in particular have been shown to overprescribe blood transfusions compared to other specialties^11–14^. The first step in dealing with a problem is acknowledging we have one. As such, the results of our study may help to raise awareness on the substantial increase in VTE risk following a blood transfusion, thereby prompting greater efforts to ration the use of transfusions and reduce VTE rates. For this purpose, Stanford Hospital recently implemented an online system that required clinicians to review the guidelines for transfusion before approving a blood transfusion order^34^. This simple reminder decreased the number of blood transfusions at the hospital by 24%, resulting in improved patient outcomes and reduced annual costs. In addition to greater awareness, additional blood management strategies should be employed for TJA with the aim of reducing the need for blood transfusion. These efforts may encompass pre-operative identification of patients at high risk for transfusion, correcting pre-operative anemia with haemopoietic agents, salvaging blood lost during the peri-operative period, limiting post-operative blood loss with haemostatic measures and individualizing transfusion triggers according to a patient’s symptoms and medical co-morbidities^35^.

Results from our regression analysis also suggested that aspirin may have a protective role in both primary and revision TJA. However, several issues prevent us from making this conclusion; first, aspirin prophylaxis has gradually become the gold standard at our institution thus more aggressive anticoagulants were most likely provided to patients that were at higher risk. Our main effort was to investigate the independent association of blood transfusion with VTE and our adjustments and matching were done accordingly thus we cannot make conclusions about the effect chemoprophylaxis agents had on VTE rates. Second, all non-aspirin prophylaxis agents were grouped under one umbrella. Dichotomization to an aspirin and non-aspirin group may introduced further bias to the independent effect aspirin has on VTE. Finally, As opposed to blood transfusion rates that remained constant during the past decade, aspirin and TXA have gradually increased together with a decrease in VTE event rates (Fig. 1). Confounders related to advancement in care such as earlier walking, same day physiotherapy and better pain control that cannot be adjusted for may have played a role. While these changes may have added to a decrease in overall VTE rates thus limiting our conclusions regarding aspirin and TXA, blood transfusion rates did not change during the course of this study therefore we believe these unmeasurable changes had a negligible effect on our conclusion about blood transfusions.

This study is not without limitations. First, this was a retrospective study which relied on data from medical records and coding for analysis and therefore conclusion making. The retrospective nature of this study may also had led to inadvertent inaccuracies in data collection as well as missing variables^36^. Due to the large scale study over a long period of time we were unable to contact patients for verification and complementary purposes. Second, VTE is effected by multiple genetic and environmental causes and many risk factors have been proposed^37–39^. While all efforts were made to capture and adjust for a comprehensive range of variables, unmeasurable variables as well as measurable variables that were not routinely recorded such as genetic predisposition and family history of VTE may have played a role in VTE development and hence confounded the results^37^. Third, despite the implementation of a nurse navigator program to track the perioperative course of all patients undergoing TJA at our institution^40^, it is possible that some VTE events may have gone undetected or unrecorded, especially if treated in an outside facility. This is especially true for DVT for which there is a higher possibility for events to either be undetected or for events to be captured without our ability to adequately assess clinical severity and this may have resulted in underestimating the association between blood transfusion and DVT as opposed to our findings on PE. Furthermore, as with previous VTE studies, event rate was relatively low effecting the magnitude of our findings^5,19^. It also prevented us from examining PE and DVT as independent outcomes in a regression analysis as the maximum likelihood estimation of the logistic model would suffer from small-sample bias^41^. Finally, in the absence of a definite institutional protocol for blood transfusions, we were unable to control for the differing indications for receiving a blood transfusions and could not comment on the necessity of transfusions recorded in this study. The use of blood transfusions at our institution was relatively low compared prior literature^11^, possibly affecting the generalizability of our results.

In conclusion, our findings show a three times higher rate of VTE in patients who receive blood transfusions following TJA. This association was mainly due to higher symptomatic PE rates. Greater efforts should be made to educate surgeons about this dangerous association and to develop perioperative protocols to reduce the need for blood transfusions in order to mitigate this risk.

## Supplementary Information

Supplementary Table 1.

Supplementary Table 2.
