# Supplementary material for: Blood transfusions increase the risk for venous thromboembolism events following total joint arthroplasty
Source: Sci Rep. 2021 Oct 28;11:21240. doi: 10.1038/s41598-021-00263-0 (PMC8553767; doi:10.1038/s41598-021-00263-0)
Supplement: Supplementary file 1 — Supplementary Table 1. [file 41598_2021_263_MOESM1_ESM.docx]

**Supplementary Table 1.**

All diagnosis (pre-admission, and in-hospital) are coded by the TJUH coding department using the ICD-9 and now ICD-10 tables.

These codes are entered into a table, making sure that pre-admission diagnosis are coded as present on admission (POA) so the can be used by us to generate the comorbidity indexes (Charlson, Elixhauser). All in-hospital diagnosis codes (non-POA) are used by us to generate the complications table.

For deep venous thrombosis (DVT) we used the following:

ICD-9

('444.2' to '444.29')

or

('451.1' to '451.29')

or

('451.84', '451.89', '451.9', '453.2', '453.3', '453.4', '453.40', '453.41', '453.42', '453.82', '453.83', '453.84', '453.85', '453.86', '453.87', '453.89', '453.9')

ICD-10

('I72.3', 'I74.2', 'I74.3', 'I80.10', 'I80.209', 'I80.3','I80.8', 'I80.9', 'I82.220', 'I82.221', 'I82.290', 'I82.3', 'I82.409', 'I82.419', 'I82.429', 'I82.439', 'I82.449', 'I82.479', 'I82.499', 'I82.4Y9', 'I82.4Z9', 'I82.609', 'I82.629', 'I82.890', 'I82.91', 'I82.A19', 'I82.B19', 'I82.C19')

For pulmonary embolism (PE) we use the following:

ICD-9

('415.1', '415.11', '415.13', '415.19')

ICD-10

('I26.90', 'I26.92', 'I26.99','T80.0XXA', 'T81.718A', 'T81.71XA', 'T81.72XA', 'T82.817A', 'T82.818A')
