# Supplementary material for: Blood transfusions increase the risk for venous thromboembolism events following total joint arthroplasty
Source: Sci Rep. 2021 Oct 28;11:21240. doi: 10.1038/s41598-021-00263-0 (PMC8553767; doi:10.1038/s41598-021-00263-0)
Supplement: Supplementary file 2 — Supplementary Table 2. [file 41598_2021_263_MOESM2_ESM.docx]

**Supplementary Table 2.** Keywords included and excluded in the search criteria to capture patients with DVT or PE.

| **INCLUDED TERMS** | **EXCLUDED TERMS** |
| --- | --- |
| BLOOD CLOT | ASPIRATION |
| CLOT | LET US KNOW |
| LEG SWELLING | FRACTURE |
| SWOLLEN LEG | MI |
| BLOOD THINNER | MYOCARDIAL INFARCT |
| CALF | INFARCT |
| PRESSURE | HEART ATTACK |
| ULTRASOUND | FALL |
| US | PAIN |
| DOPPLER | CONSTIPATION |
| VENOGRAM | NAUSEA |
| SONOGRAM | CHF |
| EMERGENCY DEPARTMENT | HEART FAILURE |
| ED | MI |
| EMERGENCY ROOM |  |
| ER |  |
| BLOOD CLOT |  |
| CLOT |  |
| LUNG |  |
| PULMONARY EMBOLISM |  |
| PE |  |
| SPIRAL CT |  |
| CT |  |
| VQ SCAN |  |
| WELLS SCORE |  |
| DIMER |  |
| CHEST X-RAY |  |
| CHEST X RAY |  |
| CHEST RADIOGRAPH |  |
| PULMONARY ANGIOGRAPHY |  |
| RAPID BREATHING |  |
| MEMOPTYSIS |  |
| SOB |  |
| SHORTNESS OF BREATH |  |
| EMERGENCY DEPARTMENT |  |
| ED |  |
| EMERGENCY ROOM |  |
| ER |  |
| CHEST PAIN |  |
| BLOOD THINNER |  |
| HEPARIN |  |
| LOW MOLECULAR WEIGHT HEPARIN |  |
| LMWH |  |
| ENOXAPARIN |  |
| COUMADIN |  |
| WARFARIN |  |
| RIVAROXABAN |  |
| XARELTO |  |
| APIXABAN |  |
| ELIQUIS |  |
| DABIGATRAN |  |
| PRADAXA |  |
| EDOXABAN |  |
| SAVAYSA |  |
